# Biodegradable Cellulose Film Prepared From Banana Pseudo-Stem Using an Ionic Liquid for Mango Preservation

**DOI:** 10.3389/fpls.2021.625878

**Published:** 2021-02-19

**Authors:** Binling Ai, Lili Zheng, Wenqi Li, Xiaoyan Zheng, Yang Yang, Dao Xiao, Jian Shi, Zhanwu Sheng

**Affiliations:** ^1^Haikou Experimental Station, Chinese Academy of Tropical Agricultural Sciences, Haikou, China; ^2^Biosystems and Agricultural Engineering, University of Kentucky, Lexington, KY, United States

**Keywords:** cellulose film, ionic liquid, banana stem fiber, mango preservation, soil burial test, biodegradability

## Abstract

The excessive use and disposal of plastic packaging materials have drawn increasing concerns from the society because of the detrimental effect on environment and ecosystems. As the most widely used fruit packing material, polyethylene (PE) film is not suitable for long-term preservation of some tropical fruits, such as mangos, due to its inferior gas permeability. Cellulose based film can be made from renewable resources and is biodegradable and environmental-friendly, which makes it a promising alternative to PE as a packaging material. In this study, cellulose film synthesized from delignified banana stem fibers *via* an ionic liquid 1-Allyl-3-methylimidazolium chloride ([AMIm][Cl]) were evaluated as packing material for mangos preservation. The moisture vapor transmission rate and gas transmission rate of the synthesized cellulose film were 1,969.1 g/(m^2^⋅24 h) and 10,015.4 ml/(m^2^⋅24 h), respectively, which are significantly higher than those of commercial PE films. The high permeability is beneficial to the release of ethylene so that contribute to extend fruit ripening period. As a result, cellulose film packaging significantly decreased the disease and color indexes of mangos, while prolonged the storage and shelf life of marketable fruits. In addition, the cellulose film was decomposed in soils in 4 weeks, indicating an excellent biodegradability as compared to the PE plastic film.

## Introduction

Plastics are widely used in food packaging applications primarily for its low costs (Kirwan et al., 2011; Mandal, 2015). Macarthur Foundation estimated in 2016 that 78 million tons of plastic packaging is produced worldwide (Axelsson and van Sebille, 2017), and an estimated 69% of which is contributed by food industry (Geueke et al., 2018). After short use (mostly single-use) as food packaging, 40% of the plastics packing materials, corresponding to 22 million tons, ends up in landfill and amasses in soil, which is a huge threat to underground water resources; while another 32%, corresponding to 17 million tons, leaks out of the collecting and sorting systems, becomes trashes and litters all over cities and oceans, and finally enters into ecosystems and food chain that is fatal to human and animal health (Guillard et al., 2018). The growing concerns on health and environment call for immediate acts worldwide to develop a sustainable and biodegradable alternative to replace plastics as packaging materials.

Additionally, as the most commonly used food packing material, polyethylene (PE) film is actually detrimental to long-term shortage of some fruits. Mango is a kind of tropical fruit, which is climacteric and ethylene-sensitive. Mangoes are typically under-ripe at harvest and ripen quite rapidly after harvest. The ripening of mangos usually takes 6–7 days at room temperature, after which the fruit quality decreases, gradually becomes diseased and rotted afterward (Su et al., 2001). Furthermore, the natural harvest season of mango is usually wet and rainy with temperatures of up to 35°C, which accelerates the deterioration and decay of mangos. Packaging is a necessary and vital step for fruit preservation, which not only provides protection from physical damage, but also keeps fruits from diseases and chemical contamination so that retains freshness and nutritional quality for long-distance transportation and prolonged storage (Mangaraj et al., 2009; Guillard et al., 2018). Film packaging with an modified atmosphere has been widely used in fruit and vegetable storage (Wilson et al., 2019). Modified atmosphere packaging maintains a balance between fruit respiration and the air permeability of packaging films, which forms a microenvironment with high CO_2_ but low O_2_ so that inhibits the metabolism of fruits and vegetables and retains freshness for extended shelf life (Dhalsamant et al., 2017; Chen et al., 2019). However, as the primary packaging materials, gas permeability of PE films is limited. Therefore, from perspective of fruit preservation, it is also necessary looking for an alternative material with enhanced moisture and vapor transmission to replace PE films for some fruits.

As one of the primary components of lignocellulosic biomass, cellulose is biodegradable and readily available, which makes it a promising alternative to plastics for food packaging applications. However, owing to strong hydrogen bonds interactions, natural cellulose possesses high orientation and crystallinity, which makes it nearly insoluble in ordinary solvents so that limits its applications. Regenerated cellulose can be obtained by chemically modifying cellulose into carboxymethyl cellulose, cellulose xanthate (Weißl et al., 2018) or a cuprammonium cellulose complex (Sayyed et al., 2019). Ionic liquids are a new category of molten salts at room temperature and certain of them have good capability for dissolving cellulose. By formation of strong hydrogen bonds with hydrogen atoms of hydroxyls in cellulose, cellulose dissolves in ionic liquids (Zhang et al., 2017). The dissolved cellulose can be easily regenerated from ionic liquid solutions via addition of water, ethanol or acetone (Zhu et al., 2006). After its regeneration, the ionic liquids can be recovered simply by vacuum evaporation and reused for several times without significant deterioration in the performance (Mai et al., 2014). Ionic liquids have been extensively investigated as cellulose-dissolving solvents to synthesize cellulose-based materials, including blends (Mundsinger et al., 2015), composites (Mahmood et al., 2017), fibers (Hummel et al., 2015), hydrogels (Xu et al., 2015), and other cellulosic materials (Isik et al., 2014; Zhang et al., 2017). Zheng et al. (2019) used ionic liquids [Bmim][Cl], [Amim][Cl] and [Emim][Ac] for dissolution of coniferous pulp and it was found that the regenerated films from [Amim][Cl] had the highest crystallinity, transparency, and tensile strength. Xu et al. (2020) reported a [Amim][Cl] based dissolution system for producing cellulose films from waste cardboard and the regenerated films had a smooth and uniform surface and high transmittance.

Banana stalk is a waste biomass after fruit harvesting produced in large volume due to each plant bearing fruit only once. The inedible parts, including pseudo-stems and leaves, representing about 88% of the weight of the whole plant (Reddy and Yang, 2015), are discarded as wastes. China alone will generate about 29.0 million tons per year of banana stalk residues (Li et al., 2016). The banana pseudo-stem has a high cellulose fiber content (Guimarães et al., 2009). Therefore, banana stem is an important yet underutilized cellulose resource, which is available to be converted into a variety of value-added products. In this study, regenerated cellulose films synthesized from delignified banana stem fibers using ionic liquid [AMIm][Cl] were evaluated as packaging material for mangos preservation. Furthermore, the biodegradation properties of the cellulose films were evaluated using a compost soil burial test. The study demonstrates a promising environmental-friendly solution to the problematic plastic packaging.

## Materials and Methods

### Materials

**FIGURE 1:**
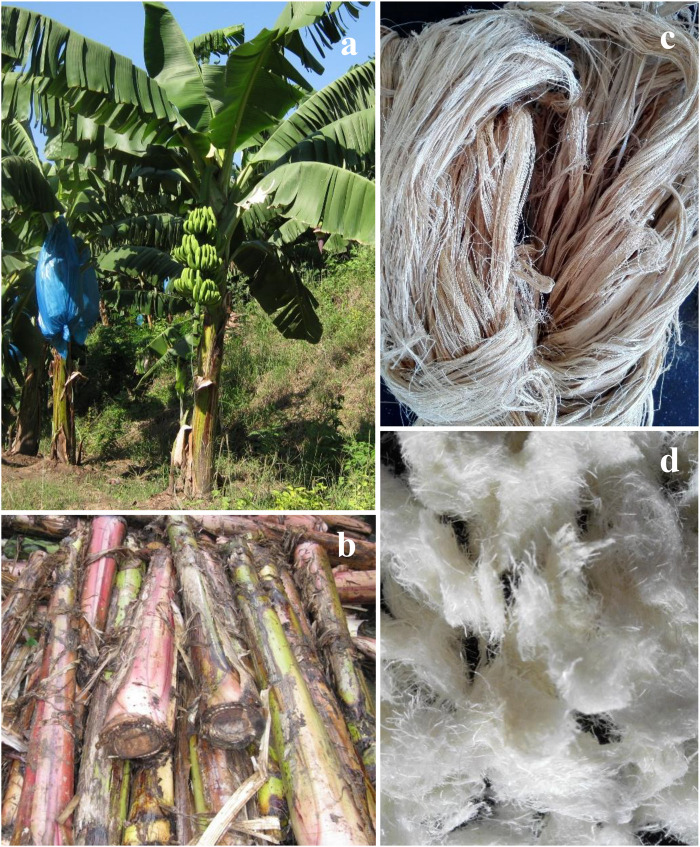
Photographs of of **(a)** banana trees with fruits and leaf sheaths, **(b)** banana stems, **(c)** banana stem fibers, and **(d)** extracted banana stem cellulose.

Banana stem fibers were obtained by scraping banana pseudo-stems, which was collected from a local banana plantation in Haikou, China. Figure 1 shows photographs of banana trees, banana pseudo-stems, banana stem fibers, and extracted cellulose by following the delignification procedure in “Delignification of Banana Stem Fibers” section. 1-Allyl-3-methylimidazolium chloride ([AMIm][Cl], purity ≥ 96%) used to dissolve extracted banana stem cellulose was purchased from Chemer Chemical Co., Ltd. (Shandong, China). Mango fruits (*Mangifera Indica* L. cv. “Xiaotainong”) were purchased from a local fruit market in Haikou, China. The fruits were just picked within a few hours and did not undergo any fresh-keeping treatment.

### Delignification of Banana Stem Fibers

The banana stem fibers (shown in Figure 1c) were soaked in H_2_SO_4_ solution (2 g/L) at 50°C for 2 h without agitation at a solid loading of 5% (w/v) to remove pectin and free sugars. The solid residue was separated by filtration and washed thoroughly with DI water until the pH value of the filtrate is neutral. The washed solid residue was then soaked in NaOH solution (200 g/L) at 30°C for 30 min to remove lignin (Shang et al., 2016). The delignified residue was thoroughly washed with DI water, squeezed to remove excess water and dried in an oven (Figure 1d). The cellulose, hemicellulose and lignin contents were measured according to detergent fiber method (Soest and Wine, 1967). Briefly, by boiling in deionized water and extracting with a neutral detergent solution the soluble fraction was removed and the neutral detergent fiber residue (NDF) was obtained. Followed by the extraction with the Van Soest acid detergent, the acid detergent fiber residue (ADF) was obtained. By extracting the ADF by sulfuric acid, the acid detergent lignin residue (ADL) was obtained. The cellulose, hemicelluloses and lignin contents are calculated as ADF–ADL, NDF–ADF, and ADL, respectively. The raw banana stem fiber contains 48.0% of cellulose, 21.1% of hemicellulose and 15.7% of lignin and the delignified residue 79.1% of cellulose, 7.6% of hemicellulose and 3.2% of lignin.

### Preparation of Cellulose Films

Extracted banana stem cellulose (1 g) and [AMIm][Cl] (19 mL) were loaded into a 50 mL round-bottom flask, and then heated in a heating mantle at 80–90°C with agitation at 200 rpm for 3–4 h. Once a transparent appearance was obtained, the mixture of dissolved cellulose and ionic liquid was cast onto a glass plate and spread with a spreader to obtain a 0.5 mm-thick layer, which was then immediately immersed in a coagulation bath (aqueous ethanol solution, 20%) to form a cellulose hydrogel. The regenerated cellulose hydrogel was then washed at least five times with distilled water to remove residual [AMIm][Cl]. The cellulose film was obtained by drying at room temperature.

### Mechanical Testing of Cellulose Films

Mechanical testing was conducting following procedures recommended in the Chinese National Standards. According to GB/T 6672-2001 (adopted from ISO 4593-1993), cellulose film thickness was determined by using a thickness gauge (CH-10-AT, Liuling Instrument, Shanghai, China) to the nearest 0.001 mm at 13 positions, and the mean values was reported. Tensile properties tests were conducted using a universal testing machine (WDT20, KQL Testing Instruments, Shenzhen, China) at a crosshead speed of 1 mm/min in accordance with the procedure specified in GB/T 1040.3-2006 (adopted from ISO 527-3:1995). Water vapor transmission were determined by sheet-cup method using a tester (W3/031, Labthink Instrument, Jinan, China) in accordance with GB/T 1037-1988 (adopted from ASTM E96-1980). The water vapor transmission (WVT) of the films was calculated by Eq. 1.

(1) $$\text{W}VT=\frac{24\times △\text{m}}{\text{A}\times \text{t}}$$

where WVT (g/m^2^⋅24 h) is the water vapor transmission rate; t (h) is the time required to reach an equilibrium (the increased weight of permeability cup is less than 5%); Δm (g) is the weight gain during the time t; A (m^2^) is the test area of the film sample.

The gas permeability was determined using an oxygen permeation analyzer (VAC-V1, Labthink Instrument, Jinan, China) in accordance with GB/T 1038-2000 (adopted from ISO 2556-1974). The gas permeability rate (GP) of the films was calculated by Eq. 2.

(2) $$\text{G}P=\frac{△\text{p}}{△\text{t}}\times \frac{\text{V}}{\text{S}}\times \frac{{\text{T}}_{0}}{{\text{p}}_{0}\text{T}}\times \frac{24}{{\text{P}}_{1}-{\text{p}}_{2}}$$

Where GP (ml/m^2^⋅24 h) is the oxygen permeability rate; Δp/Δt (Pa/h) is the average value of the pressure change of low-pressure chamber in unit time after oxygen permeation is stable; V (cm^3^) is the volume of the low-pressure chamber; T_0_ is standard temperature (273.15 K); S (m^2^) is the test area of the film sample; p_0_ is standard pressure (1.01 × 10^5^ Pa); T (K) is the test temperature; p_1_–p_2_ (Pa) is the pressure difference between the two sides of film sample.

### Characterization Analysis of Cellulose Films

The morphology of cellulosic materials was monitored using a scanning electron microscope (SEM) (S-3000N, Hitachi). Fourier-transform infrared (FTIR) spectra were obtained using an FTIR spectrophotometer (Tensor27, Bruker) by mixing ground samples with KBr to prepare pellets. FTIR spectra were recorded in the spectral range of 400–4,000 cm^–1^. Thermogravimetric analysis (TGA) was performed using a thermal analyzer (SDT Q600, TA Instruments) under a nitrogen atmosphere by increasing the heating temperature up to 800°C at a linear rate of 10°C/min. X-ray diffraction (XRD) analysis was performed using X-ray powder diffractometer (D8-Advance, Bruker). Samples were scanned in the 2q range of 10–30°.

### Preservation of Mango Using Cellulose Films

Two sheets of cellulose film were glued together to fabricate a packaging bag (approximately 10 × 10 cm in size). Plastic packaging bags of the same size were also prepared from commercially available polyethylene wrap. The mango fruits were purchased from a local fruit market without any preservation treatment. Fruits of similar shape and size, and without visible mechanical and pathological damage, were selected for preservation experiments. The selected fruits were washed with tap water for 1 min, dried with absorbent paper, and then individually packed into cellulose or polyethylene film bags, and stored at room temperature or kept in an incubator at 11°C. Each group consisted of at least 10 fruits. The color index, disease index, diseased fruit rate, marketable fruit rate, and weight loss ratio were calculated on days 7 and 14.

The color index for recording the process of fruit peel degreening was calculated using equation (3) (Gong et al., 1994).

(3) $$\mathrm{Color}\mathrm{index}=\sum \frac{\mathrm{color}\mathrm{score}\times \mathrm{Fruit}\mathrm{number}}{\mathrm{The}\mathrm{highest}\mathrm{color}\mathrm{score}\times \mathrm{Total}\mathrm{number}\mathrm{of}\mathrm{fruits}}\times 100$$

where the following scoring system was adopted: 0 points for all green; 1 point for a yellow fruit pedicel; 2 point for localized parts turning yellow; 3 points for most parts turning yellow; 4 points for all yellow.

The disease index was evaluated by assessing the total decayed area using Eq. (4) (Gong et al., 1994).

(4) $$\mathrm{Disease index}=\sum \frac{\mathrm{Disease}\mathrm{score}\times \mathrm{Fruit}\mathrm{number}}{\begin{array}{c} \mathrm{The}\mathrm{highest}\mathrm{Disease}\mathrm{score}\times \mathrm{Total}\mathrm{number}\mathrm{of}\mathrm{fruits} \end{array}}\times 100$$

where the following scoring system was adopted: 0 points for no visible decay; 1 point for less than 1% decay spots; 2 points for less than 20% decay spots; 3 points for more than 20%, but less than 50% decay spots; 4 points for more than 50% decay spots.

Fruits with disease scores of 0 and 1 have commercial value. The total percentage of fruits with these scores was defined as the marketable fruit rate. The total percentage of fruits with scores of 1–4 is defined as the diseased fruit rate. The percentage cumulative weight loss of fruits is defined as the weight loss ratio.

### Biodegradability Evaluation of Cellulose Films

The soil burial test was conducted with reference to similar methods (Rudnik and Briassoulis, 2011; Gautam and Kaur, 2013; Maran et al., 2014). This degradation test was conducted using natural microorganisms, which better reflected the degradation of biomaterials in natural environments. To facilitate the removal of samples from the soil, the polyethylene film, cellulose film, and quantitative filter paper (Xinxing 202, Hangzhou Special Paper Factory, Hangzhou, China) were clamped between two pieces of nylon mesh. A container was filled with a 10 cm-thick soil layer, samples were tiled with nylon mesh on the soil layer, and then covered with another 10 cm-thick soil layer. The soil moisture was maintained at approximate 50%. Samples were taken each week, and the attached soils were carefully rinsed with deionized water. The constant weight of each sample was measured before and after degradation and the weight loss of each sample was calculated.

### Statistical Analysis

Tests for statistical significance were performed using Statistical Product and Service Solutions (IBM SPSS Statistics for Windows, Version 19.0, IBM Corp., New York, United States).

## Results and Discussion

### Mechanical and Physicochemical Properties of Cellulose Films

The intensity of ionic liquid processing significantly affects the properties of the banana stem-derived cellulose film. When the banana stem fiber-derived cellulose was treated in [AMIm][Cl] at 90°C for 3 h, the resulting cellulose film was transparent and exhibited a tensile strength of 32.8 MPa. As the intensity of treatment decrease to 80°C for 4 h, the prepared cellulose film had an obviously improved tensile strength of 77.0 MPa. However, it turns to be translucent, probably due to incomplete dissolution of cellulose. The mechanical properties of cellulose films acquired through [AMIm][Cl] treatment under different operation conditions are shown in Table 1.

**TABLE 1:** Mechanical properties of cellulose films.

Test items	Transparent cellulose film (90°C, 3 h)	Translucent cellulose film (80°C, 4 h)	Polyethylene film (Yaptenco et al., 2007)
Thickness, μm	21.1 ± 2.4 A^#^	23.1 ± 0.5 A	25.1 ± 0.6
Tensile strength, MPa	32.8 ± 7.2 A	77.0 ± 2.5 B	N/A
Extension at break, %	4.0 ± 0.5 A	3.1 ± 0.4 A	N/A
Water vapor transmission rate, g/(m^2^⋅24 h)	1,969.1 ± 88.5 A	1,765.9 ± 23.4 A	N/A
Gas permeability rate, g/(m^2^⋅24 h)	10,015.4 ± 1,117.1 A	9,325.8 ± 526.9 A	5,703.9–8,526.1

*^#^Values are expressed as mean ± SEM (n = 3). Values in the same row followed by the same uppercase letter are not significantly different at P = 0.05, according to Duncan’s multiple range test.*

The prepared cellulose films possess an enhanced permeability. The moisture vapor transmission rate and gas transmission rate are measured to be 1,969.1 g/(m^2^⋅24 h) and 10,015.4 ml/(m^2^⋅24 h), respectively, which were significantly higher than those of traditional polyethylene films (Yaptenco et al., 2007; Table 1). The high permeability of cellulose film is beneficial to ethylene release in the modified atmosphere packaging of fruits and vegetables, which can delay the postharvest ripening of climacteric fruits, such as mango, resulting in an extended storage period.

**FIGURE 2:**
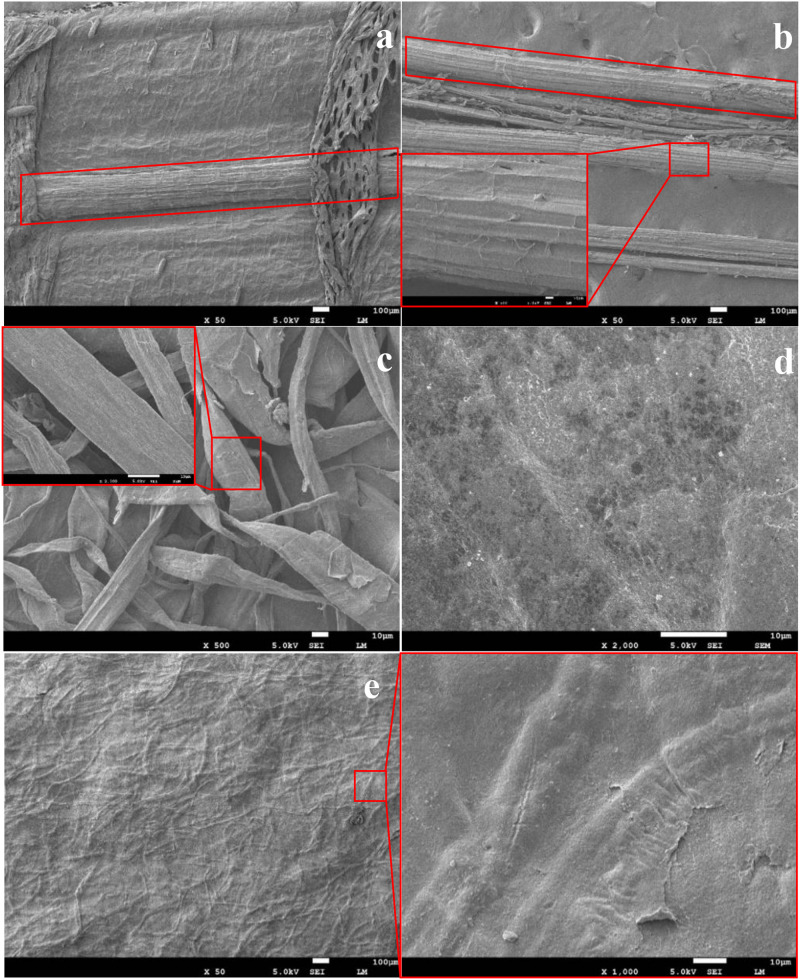
SEM images of **(a)** untreated banana stem, **(b)** banana stem fiber, **(c)** extracted banana stem cellulose, **(d)** transparent cellulose film (90°C, 3 h), and **(e)** translucent cellulose film (80°C, 4 h).

The morphology changes associated with the banana stem fiber derived cellulose film can be observed from SEM images, as shown in Figure 2. Fiber bundles were separated from the banana pseudo-stem by mechanical scraping (Figure 2a) to obtain banana pseudo-stem fiber (Figure 2b). The images show that no obvious change was observed in the fiber morphology during mechanical scraping, but the fiber bundles became loose and irregular (Figure 2c) after removing lignin fraction. Cellulose film (Figure 2d) prepared from alkaline-delignified cellulose fiber by ionic liquid dissolution (at 90°C for 3 h) had a smooth surface without obvious fibrous structure. While in the translucent cellulose film, as can be seen from Figure 2e, the cellulose fibers were not completely dissolved, and the tiny fibers were intertwined in a network structure. As mentioned above, the mechanical strength of the translucent cellulose films was higher than that of the transparent cellulose films, probably due to the incomplete dissolution of cellulose fibers that resulted in a homogeneous mixture of microfibers and nanofibers. In a micro- and nano-hybrid cellulose film, nanofibers fill the voids among neighboring microfibers, making the hybrid film densely packed, and hence a higher mechanical strength (Wang et al., 2020).

**FIGURE 3:**
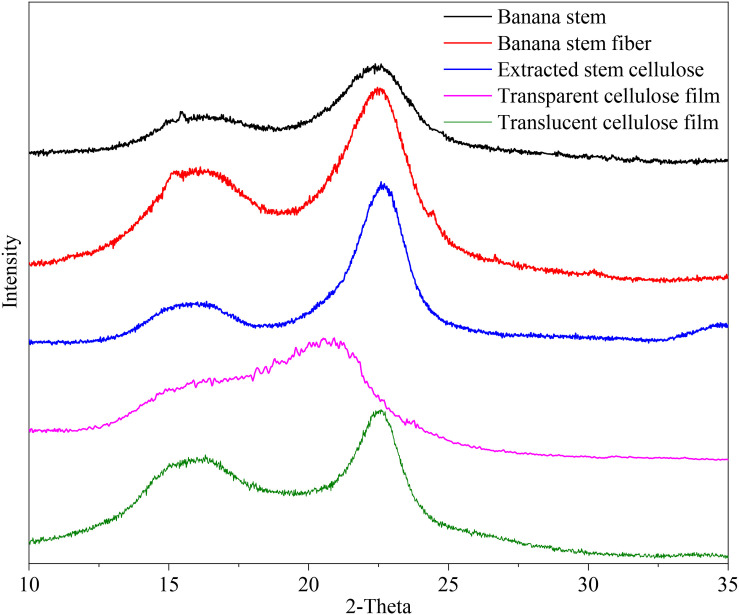
X-ray diffraction profiles of banana stem, banana stem fiber, extracted banana stem cellulose, transparent cellulose film (90°C, 3 h), and translucent cellulose film (80°C, 4 h).

The XRD profiles of banana stem, stem fiber and extracted cellulose show well-defined diffraction peaks at 16.4° and 22.6°, while, clearly, that of transparent cellulose film exhibits only a broad peak at 20.3° (Figure 3). This broad peak is considered an overlapped peak of two peaks at 20.1° and 21.9° and the peak of amorphous cellulose at 17.3° (Xia et al., 2016), indicating that after ionic liquid dissolution and regeneration, type I cellulose was transformed into type II cellulose along with a large number of amorphous cellulose structures (Cheng et al., 2012). The XRD profile of translucent cellulose film is quite different from that of transparent film. The translucent cellulose film maintained the crystal structure of type I cellulose, but the diffraction peak at 22.6° had a relatively low value, indicating that part of type I cellulose transformed to type II cellulose and amorphous cellulose. This XRD result is in conformity with the SEM observation that only part of cellulose fibers was dissolved by ionic liquid when preparing translucent cellulose films. Transparency is a necessary characteristic of cellulose film, so, unless otherwise specified, the cellulose film mentioned below refers to a transparent cellulose film.

### Application of Cellulose Films in Mango Fresh-Keeping

Freshness is one of the most important qualities of fruits. Fresh-keeping packaging requires good moisture-resistance, mechanical strength, and heat and mass transfer properties, which are necessary to keep the fruits hydrated and intact, and transfer heat and gas produced by the physiological activities of fruits (Soltani et al., 2015; Zhang et al., 2016). The cellulose film has high moisture vapor transmission rate and gas transmission rate, which is good for humidity diffusion, heat dissipation and ethylene release. A high humidity results in water condensation on the surface of both film and fruits, and the in-pack condensation leads to a rapid decay (Aharoni et al., 2007). The internal heat generation from respiration and accumulation of ethylene produced by autocatalytic ethylene synthesis accelerate the ripening and even decay of fruits (Blanke, 2014). Therefore, cellulose film with high permeability could be suitable for mango preservation and storage.

**FIGURE 4:**
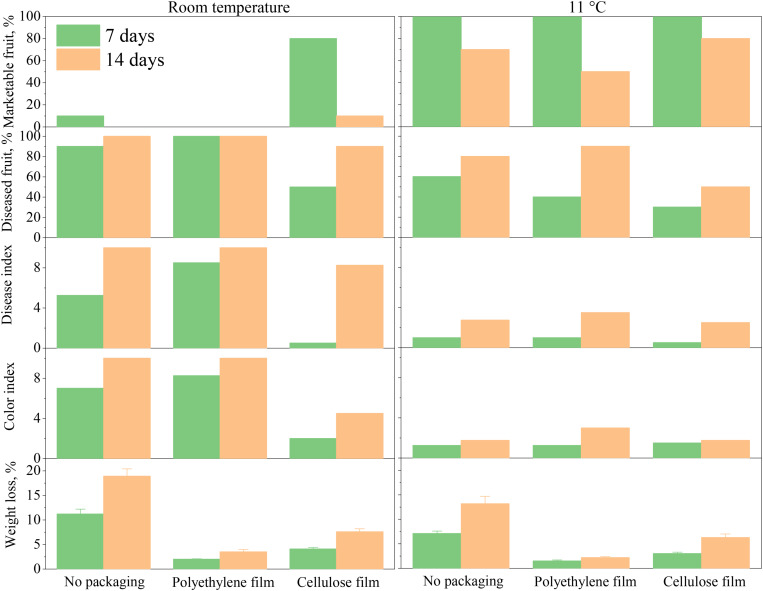
Comparison of fresh-keeping effects of cellulose film and polyethylene film on mango storage at room temperature (left) and 11°C (right).

Figure 4 and Supplementary Table S1 demonstrates the effectiveness of cellulose film on mongo fresh-keeping compared to PE film. As shown in Figure 4, polyethylene film packaging accelerated the deterioration and decay of mangoes. At room temperature, the disease indexes of mango packaged with polyethylene film was 8.5 at 7 days, which was higher than that without packaging (5.25). At 11°C, the disease indexes of mango packaged with polyethylene film was 3.5 at 14 days, which was higher than that without packaging (2.75). When cellulose film packaging was used, the disease index was significantly reduced, and the marketable fruit rate significantly increased. When stored at room temperature for 7 days, the marketable fruit rate of cellulose film packaging was 80%, while those of polyethylene film-packaged and unpackaged samples were 0 and 10%, respectively. Cellulose film packaging also slowed the pericarp yellowing rate. When stored at room temperature for 7 days, the color index of cellulose film-packaged pericarp was 0.5, while those of polyethylene film-packaged and unpackaged pericarp were higher, at 8.5 and 5.25, respectively. Polyethylene is the most dominant packaging material used in fruit preservation, but not in mango preservation. Owing to the low tolerance of mango to CO_2_, sealed preservation using polyethylene film can accelerate mango fruit deterioration. For example, when the CO_2_ concentration was higher than 8% and the O_2_ concentration was less than 2%, the color of mango pericarp turned gray and the flavor was lost (Zong, 2007).

An appropriate low temperature can effectively inhibit the respiration intensity and delay the occurrence of fruit ripening and disease (Coates and Johnson, 1997). Mango as a climacteric fruit has a very short shelf life. The shelf life ranges from 4 to 8 days at room temperature, while it can be extended to 2–3 weeks in cold storage at 13°C (Carrillo-Lopez et al., 2000). As can be seen from Figure 4, low temperature storage can significantly inhibit the occurrence of diseases and pericarp yellowing, increase the marketable fruit rate, and extend the storage life. When stored at 11°C, the effect of packaging materials on mango preservation was consistent with that of storage at room temperature, with cellulose film packaging able to further improve the fruit commodity rate and extend the storage period. However, lower temperatures cannot further extend the storage life, on the contrary, it will cause chilling injury (Phakawatmongkol et al., 2004). The chilling injury symptoms include skin browning and pulp discoloration. The minimum tolerance temperatures of mango varied among cultivars, ranging from 10 to 13°C (Phakawatmongkol et al., 2004; Yan et al., 2014).

According to Liu et al., in China the annual loss of fresh fruits and vegetables in transportation is 10–20%, that is, every year nearly 14 million tons of fruits and 100 million tons of vegetables are wasted in transit from farm to market (Liu, 2014). The loss rate of fruits and vegetables in developed countries is controlled within 5% (Hailu and Derbew, 2015), because of the well-established cold chain facilities and infrastructure. The overall coverage rate of cold chain logistics of fruits and vegetables in Europe and US has reached more than 95%, while that in China in 2015 is merely 19% (Hu et al., 2019). Cold chain management is a key player in the maintenance of the quality and minimization of the loss of fresh fruits and vegetables (Mercier et al., 2017). As the results shown in Figure 4, low-temperature storage effectively maintained mango quality and significantly reduced the loss, which has a greater impact than packaging materials. However, at room temperature storage conditions, where cold storage infrastructure is scare, cellulose film packaging can help to improve the fruit commodity rate and extend the storage period.

### Biodegradability of Cellulose Films

Disposable plastics are widely used in fruit packaging, but it might take hundreds of years to be decomposed in landfills. Andrady (1998) calculated that only 0.1% per year of the carbon of PE polymer is converted into CO_2_ by biodegradation under best laboratory exposure condition. In natural environment the degradation processes are even slower, as the conditions are not optimized for polymer degradation (Gewert et al., 2015).

**FIGURE 5:**
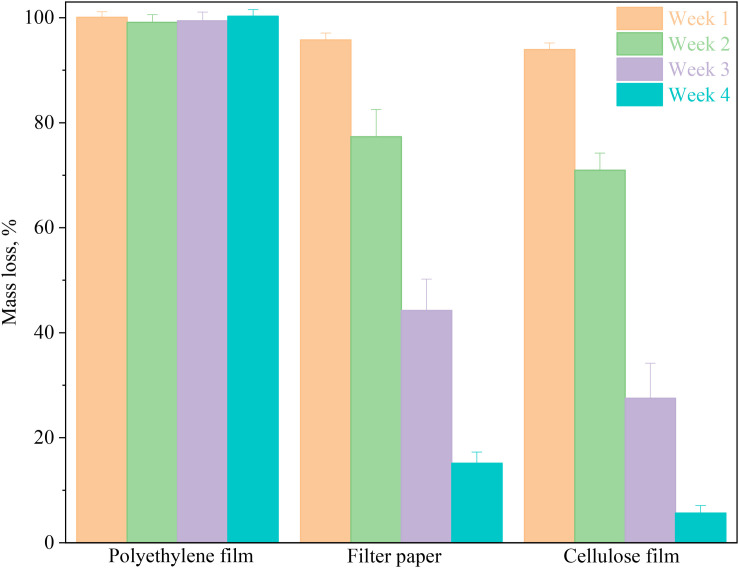
Weight changes during 4 weeks periods in soil-buried cellulose film, polyethylene film, and filter paper.

As a biomass-based material, the cellulose film exhibited an excellent degradation property (Figure 5 and Supplementary Table S2). The mass loss of cellulose film and filter paper in the early stage of degradation was low, while the mass loss rate in the later stage were accelerated. In the first week, about 4 and 6% by weight of filter paper and cellulose film was lost; in the following 2 weeks, filter paper lost 17 and 22% of its weight each week, respectively, and cellulose film 25 and 31%, respectively. After 4 weeks of simulated natural degradation in soil, the mass residual rates of the cellulose film and filter paper were 7 and 15%, respectively. Cellulose had a higher crystallinity in the filter paper, while the crystallinity of the cellulose film was decreased during ionic liquid dissolution. Moreover, the specific surface area and number of hydrophilic groups increased. These factors make the cellulose film more vulnerable to soil microorganisms (Mohan, 2011), resulting in the fast degradation of cellulose film when compared with filter paper.

**FIGURE 6:**
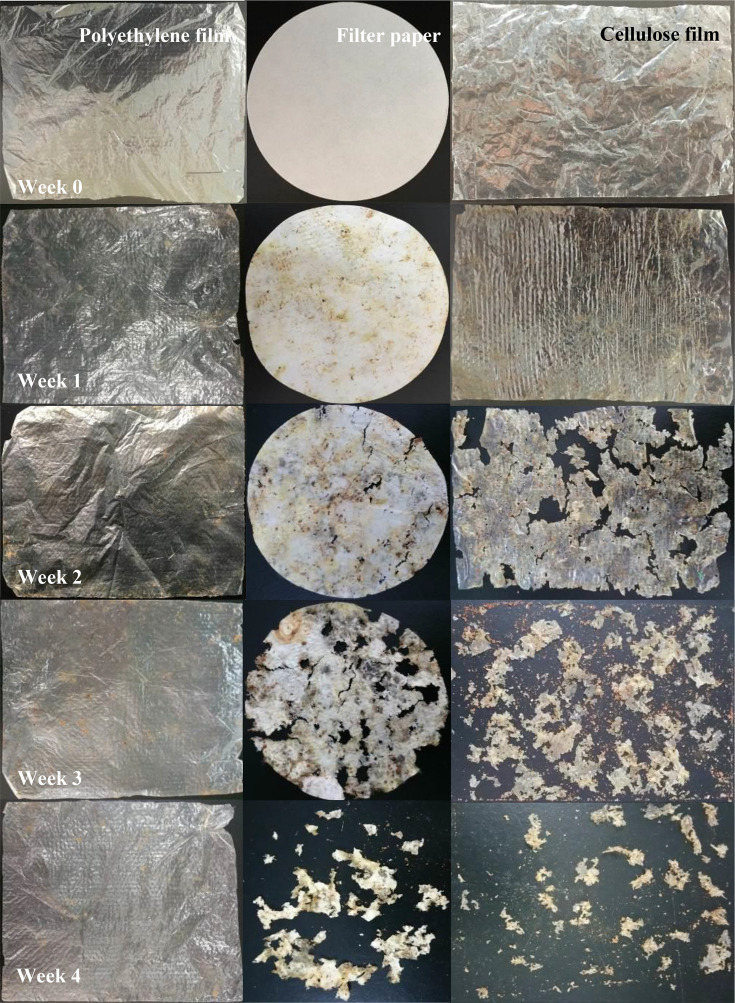
Morphology changes of polyethylene film, filter paper, and cellulose film at different soil-burying stages.

As shown by the morphological characteristics of the soil-buried samples (Figure 6), yellow/brown spots appeared on the surface of the filter paper and cellulose film after the first week of burying in soil, as characterized by microbial infection (Zorec et al., 2014). After the second week of burying, obvious etches, holes, and cracks appeared on the filter paper surface, while the cellulose film degraded faster and became fragmented. After the third week of burying, the holes on the filter paper surface were further expanded, while the cellulose film was completely fragmented. After the fourth week of burying in soil, the filter paper was completely fragmented, while the cellulose film showed only flocculent residues.

The soil burial method and activated sludge method for testing biodegradability of biomaterials use microorganisms existing in the natural environment to degrade tested polymers. The advantage of these methods is that they simulate *in situ* condition (Itävaara and Vikman, 1996). However, they have poor repeatability caused by soil and activated sludge sources, season, environmental conditions, and are not suitable for determining decomposition products. The methods using specific microorganisms and enzymes are designed to determine the inherent biodegradability of biopolymers under optimal controlled conditions. These methods may not be necessarily representative of any specific environmental conditions but they ensure repeatability (Briassoulis and Degli Innocenti, 2017). Good methods reflecting the actual process of polymer biodegradation in nature with good repeatability and reproducibility still need to be investigated.

Biodegradable films for fruits and vegetables packaging have been commercially available, derived from various biopolymers including poly(lactic acid), starch, and polyhydroxyalkanoates (Abdul Khalil et al., 2018). In present study, commercial implementation of cellulose-based film are facing many challenges. Cellulose film possesses poor mechanical properties as compared to its commercial competitors. Various additives has been proposed to use to improve the film characteristics (Abdul Khalil et al., 2018). Also, the benefits gained from the low cost of lignocellulosic feedstock is completely counteracted by the cost of ILs. Moreover, harsh solvents such as H_2_SO_4_ and NaOH were used in the processes of lignin removal and cellulose extraction, which would cause secondary pollution. Still much work is needed to be done to make the manufacturing process green. Although it is just impossible to completely replace synthetic plastics with biomaterials and it may be even unnecessary, the use of biodegradable packaging materials should be the future (Tharanathan, 2003; Siracusa et al., 2008).

## Conclusion

Cellulose films were prepared from banana stem fibers by ionic liquid dissolution. The moisture vapor transmission rate and gas transmission rate were 1,969.1 g/(m^2^⋅24 h) and 10,015.4 ml/(m^2^⋅24 h), respectively, which were significantly higher than those of commercial polyethylene films. The high permeability of cellulose film was beneficial to ethylene release, resulting in delayed fruit ripening. Cellulose film packaging decreased the disease indexes of mango, increased the marketable fruit rates, and decreased the color indexes, which could extend the storage and shelf life at room temperature. The mass residual rate of cellulose film was 7.0% after 4 weeks of burying in soil, indicating that cellulose film has excellent biodegradability. This study provides a method for preparing cellulose films from low-grade cellulosic resources as promising packaging materials for fresh-keeping tropical fruits.

## Data Availability Statement

The original contributions presented in the study are included in the article/Supplementary Material, further inquiries can be directed to the corresponding author/s.

## Author Contributions

BA: funding acquisition, project administration, conceptualization, resources, methodology, investigation, data curation, visualization, formal analysis, writing—original draft, and writing—review and editing. LZ: investigation, data curation, and writing—original draft. WL: writing—original draft and writing—review and editing. XZ, YY, and DX: investigation. JS: funding acquisition, writing—original draft, and writing—review and editing. ZS: funding acquisition, resources, methodology, investigation, writing—original draft, and writing—review and editing. All authors contributed to the article and approved the submitted version.

## Conflict of Interest

The authors declare that the research was conducted in the absence of any commercial or financial relationships that could be construed as a potential conflict of interest. The handling editor declared a past co-authorship with the authors BA, WL, and JS.

## References

[B1] Abdul Khalil H. P. S. Banerjee A. Saurabh C. K. Tye Y. Y. Suriani A. B. Mohamed A. (2018). Biodegradable films for fruits and vegetables packaging application: preparation and properties. *Food Eng. Rev.* 10 139–153. 10.1007/s12393-018-9180-3

[B2] Aharoni N. Rodov V. Fallik E. Afek U. Chalupowicz D. Aharon Z. (2007). “Modified atmosphere packaging for vegetable crops using high-watervapor-permeable films,” in *Intelligent and Active Packaging for Fruits and Vegetables*, ed. Wilson C. L. (New York: CRC Press Taylor&Francis Group), 73–112. 10.1201/9781420008678.ch5

[B3] Andrady A. L. (1998). “Biodegradation of plastics: monitoring what happens,” in *Plastics Additives*, ed. Pritchard G. (Dordrecht: Springer), 32–40. 10.1007/978-94-011-5862-6_5

[B4] Axelsson C. van Sebille E. (2017). Prevention through policy: urban macroplastic leakages to the marine environment during extreme rainfall events. *Mar. Pollut. Bull.* 124 211–227. 10.1016/j.marpolbul.2017.07.024 28755809 PMC5667635

[B5] Blanke M. M. (2014). Reducing ethylene levels along the food supply chain: a key to reducing food waste? *J. Sci. Food Agric.* 94 2357–2361. 10.1002/jsfa.6660 24648006

[B6] Briassoulis D. Degli Innocenti F. (2017). “Standards for soil biodegradable plastics,” in *Soil Degradable Bioplastics for a Sustainable Modern Agriculture*, ed. Malinconico M. (Berlin: Springer), 139–168. 10.1007/978-3-662-54130-2_6

[B7] Carrillo-Lopez A. Ramirez-Bustamante F. Valdez-Torres J. Rojas-Villegas R. Yahia E. (2000). Ripening and quality changes in mango fruit as affected by coating with an edible film. *J. Food Qual.* 23 479–486. 10.1111/j.1745-4557.2000.tb00573.x

[B8] Chen J. Hu Y. Yan R. Hu H. Chen Y. Zhang N. (2019). Modeling the dynamic changes in O2 and CO2 concentrations in MAP-packaged fresh-cut garlic scapes. *Food Packag. Shelf Life* 22:100432. 10.1016/j.fpsl.2019.100432

[B9] Cheng G. Varanasi P. Arora R. Stavila V. Simmons B. A. Kent M. S. (2012). Impact of ionic liquid pretreatment conditions on cellulose crystalline structure using 1-ethyl-3-methylimidazolium acetate. *J. Phys. Chem. B* 116 10049–10054. 10.1021/jp304538v 22823503

[B10] Coates L. Johnson G. (1997). “Postharvest diseases of fruit and vegetables,” in *Plant Pathogens and Plant Diseases*, eds Brown J. Ogle H. (Burlington, MA: Rockvale Publications), 533–548.

[B11] Dhalsamant K. Mangaraj S. Bal L. M. (2017). Modified atmosphere packaging for mango and tomato: an appraisal to improve shelf life. *J. Packag. Technol. Res.* 1 127–133. 10.1007/s41783-017-0021-2

[B12] Gautam N. Kaur I. (2013). Soil burial biodegradation studies of starch grafted polyethylene and identification of *Rhizobium meliloti* therefrom. *J. Environ. Chem. Ecotoxicol.* 5 147–158.

[B13] Geueke B. Groh K. Muncke J. (2018). Food packaging in the circular economy: overview of chemical safety aspects for commonly used materials. *J. Clean. Prod.* 193 491–505. 10.1016/j.jclepro.2018.05.005

[B14] Gewert B. Plassmann M. M. Macleod M. (2015). Pathways for degradation of plastic polymers floating in the marine environment. *Environ. Sci. Process. Impacts* 17 1513–1521. 10.1039/c5em00207a 26216708

[B15] Gong G. Chen Y. Zhou S. (1994). Studies on room temperature storage of mango. *Sci. Agric. Sin.* 27 82–88.

[B16] Guillard V. Gaucel S. Fornaciari C. Angellier-Coussy H. Buche P. Gontard N. (2018). The next generation of sustainable food packaging to preserve our environment in a circular economy context. *Front. Nutr.* 5:121. 10.3389/fnut.2018.00121 30564581 PMC6288173

[B17] Guimarães J. Frollini E. Da Silva C. Wypych F. Satyanarayana K. (2009). Characterization of banana, sugarcane bagasse and sponge gourd fibers of Brazil. *Ind. Crops Prod.* 30 407–415. 10.1016/j.indcrop.2009.07.013

[B18] Hailu G. Derbew B. (2015). Extent, causes and reduction strategies of postharvest losses of fresh fruits and vegetables– a review. *J. Biol. Agric. Healthc.* 5 49–64.

[B19] Hu G. Mu X. Xu M. Miller S. A. (2019). Potentials of GHG emission reductions from cold chain systems: case studies of China and the United States. *J. Clean. Prod.* 239:118053. 10.1016/j.jclepro.2019.118053

[B20] Hummel M. Michud A. Tanttu M. Asaadi S. Ma Y. Hauru L. K. (2015). “Ionic liquids for the production of man-made cellulosic fibers: opportunities and challenges,” in *Cellulose Chemistry and Properties: Fibers, Nanocelluloses and Advanced Materials*, ed. Rojas O. (Cham: Springer), 133–168. 10.1007/12_2015_307

[B21] Isik M. Sardon H. Mecerreyes D. (2014). Ionic liquids and cellulose: dissolution, chemical modification and preparation of new cellulosic materials. *Int. J. Mol. Sci.* 15 11922–11940. 10.3390/ijms150711922 25000264 PMC4139821

[B22] Itävaara M. Vikman M. (1996). An overview of methods for biodegradability testing of biopolymers and packaging materials. *J. Environ. Polym. Degrad.* 4 29–36. 10.1007/bf02083880

[B23] Kirwan M. J. Plant S. Strawbridge J. W. (2011). “Plastics in food packaging,” in *Food and Beverage Packaging Technology*, eds Coles R. Kirwan M. (Oxford: Wiley-Blackwell), 157–212. 10.1002/9781444392180.ch7

[B24] Li C. Liu G. Nges I. A. Deng L. Nistor M. Liu J. (2016). Fresh banana pseudo-stems as a tropical lignocellulosic feedstock for methane production. *Energy Sustain. Soc.* 6:27.

[B25] Liu G. (2014). “Food losses and food waste in China: a first estimate,” in *OECD Food, Agriculture and Fisheries Papers* (Paris: OECD Publishing), 1–29. 10.1016/b978-0-12-815357-4.00001-8

[B26] Mahmood H. Moniruzzaman M. Yusup S. Welton T. (2017). Ionic liquids assisted processing of renewable resources for the fabrication of biodegradable composite materials. *Green Chem.* 19 2051–2075. 10.1039/c7gc00318h

[B27] Mai N. L. Ahn K. Koo Y.-M. (2014). Methods for recovery of ionic liquids—a review. *Process Biochem.* 49 872–881. 10.1016/j.procbio.2014.01.016

[B28] Mandal G. (2015). “Value addition of fruits and vegetables through packaging,” in *Value Addition of Horticultural Crops: Recent Trends and Future Directions*, eds Sharangi A. Datta S. (New Delhi: Springer), 191–199. 10.1007/978-81-322-2262-0_11

[B29] Mangaraj S. Goswami T. K. Mahajan P. V. (2009). Applications of plastic films for modified atmosphere packaging of fruits and vegetables: a review. *Food Eng. Rev.* 1 133–158. 10.1007/s12393-009-9007-3

[B30] Maran J. P. Sivakumar V. Thirugnanasambandham K. Sridhar R. (2014). Degradation behavior of biocomposites based on cassava starch buried under indoor soil conditions. *Carbohydr. Polym.* 101 20–28. 10.1016/j.carbpol.2013.08.080 24299744

[B31] Mercier S. Villeneuve S. Mondor M. Uysal I. (2017). Time–temperature management along the food cold chain: a review of recent developments. *Compr. Rev. Food Sci. Food Safety* 16 647–667. 10.1111/1541-4337.12269 33371570

[B32] Mohan K. (2011). Microbial deterioration and degradation of polymeric materials. *J. Biochem. Technol.* 2 210–215.

[B33] Mundsinger K. Müller A. Beyer R. Hermanutz F. Buchmeiser M. R. (2015). Multifilament cellulose/chitin blend yarn spun from ionic liquids. *Carbohydr. Polym.* 131 34–40. 10.1016/j.carbpol.2015.05.065 26256157

[B34] Phakawatmongkol W. Ketsa S. Van Doorn W. G. (2004). Variation in fruit chilling injury among mango cultivars. *Postharvest Biol. Technol.* 32 115–118. 10.1016/j.postharvbio.2003.11.011

[B35] Reddy N. Yang Y. (2015). “Fibers from banana pseudo-stems,” in *Innovative Biofibers from Renewable Resources* (Berlin: Springer), 25–27. 10.1007/978-3-662-45136-6_7

[B36] Rudnik E. Briassoulis D. (2011). Degradation behaviour of poly (lactic acid) films and fibres in soil under mediterranean field conditions and laboratory simulations testing. *Ind. Crops Prod.* 33 648–658. 10.1016/j.indcrop.2010.12.031

[B37] Sayyed A. J. Deshmukh N. A. Pinjari D. V. (2019). A critical review of manufacturing processes used in regenerated cellulosic fibres: viscose, cellulose acetate, cuprammonium, LiCl/DMAc, ionic liquids, and NMMO based lyocell. *Cellulose* 26 2913–2940. 10.1007/s10570-019-02318-y

[B38] Shang W. Sheng Z. Shen Y. Ai B. Zheng L. Yang J. (2016). Study on oil absorbency of succinic anhydride modified banana cellulose in ionic liquid. *Carbohydr. Polym.* 141 135–142. 10.1016/j.carbpol.2016.01.009 26877005

[B39] Siracusa V. Rocculi P. Romani S. Rosa M. D. (2008). Biodegradable polymers for food packaging: a review. *Trends Food Sci. Technol.* 19 634–643.

[B40] Soest P. V. Wine R. (1967). Use of detergents in the analysis of fibrous feeds. IV. Determination of plant cell-wall constituents. *J. Assoc. Off. Anal. Chem.* 50 50–55. 10.1093/jaoac/50.1.50

[B41] Soltani M. Alimardani R. Mobli H. Mohtasebi S. S. (2015). Modified atmosphere packaging: a progressive technology for shelf-life extension of fruits and vegetables. *J. Appl. Packag. Res.* 7 33–59.

[B42] Su X. Jiang Y. Yu X. He S. (2001). Review on postharvest biology and technology for storage and transport of mango fruit. *J. Zhongkai Agrotechnical Coll.* 14 60–66.

[B43] Tharanathan R. N. (2003). Biodegradable films and composite coatings: past, present and future. *Trends Food Sci. Technol.* 14 71–78. 10.1016/s0924-2244(02)00280-7

[B44] Wang X. Pang Z. Chen C. Xia Q. Zhou Y. Jing S. (2020). All-Natural, degradable, rolled-up straws based on cellulose micro-and nano-hybrid fibers. *Adv. Funct. Mater.* 30:1910417. 10.1002/adfm.201910417

[B45] Weißl M. Niegelhell K. Reishofer D. Zankel A. Innerlohinger J. Spirk S. (2018). Homogeneous cellulose thin films by regeneration of cellulose xanthate: properties and characterization. *Cellulose* 25 711–721. 10.1007/s10570-017-1576-3

[B46] Wilson M. D. Stanley R. A. Eyles A. Ross T. (2019). Innovative processes and technologies for modified atmosphere packaging of fresh and fresh-cut fruits and vegetables. *Crit. Rev. Food Sci. Nutr.* 59 411–422. 28891686 10.1080/10408398.2017.1375892

[B47] Xia G. Wan J. Zhang J. Zhang X. Xu L. Wu J. (2016). Cellulose-based films prepared directly from waste newspapers via an ionic liquid. *Carbohydr. Polym.* 151 223–229. 10.1016/j.carbpol.2016.05.080 27474561

[B48] Xu H. Huang L. Xu M. Qi M. Yi T. Mo Q. (2020). Preparation and properties of cellulose-based films regenerated from waste corrugated cardboards using [Amim] Cl/CaCl2. *ACS Omega* 5 23743–23754. 10.1021/acsomega.0c02713 32984693 PMC7513365

[B49] Xu M. Huang Q. Wang X. Sun R. (2015). Highly tough cellulose/graphene composite hydrogels prepared from ionic liquids. *Ind. Crops Prod.* 70 56–63. 10.1016/j.indcrop.2015.03.004

[B50] Yan M. Huang J. Lin X. Zeng H. Li X. (2014). Research progress on storage technology of mango. *Acad. Period. Farm Prod. Process.* 9 72–76.

[B51] Yaptenco K. F. Kim J. G. Lim B. S. (2007). Gas transmission rates of commercially available polyethylene and polypropylene films for modified atmosphere packaging. *Philipp. Agric. Sci.* 90 22–27.

[B52] Zhang J. Wu J. Yu J. Zhang X. He J. Zhang J. (2017). Application of ionic liquids for dissolving cellulose and fabricating cellulose-based materials: state of the art and future trends. *Mater. Chem. Front.* 1 1273–1290. 10.1039/C6QM00348F

[B53] Zhang M. Meng X. Bhandari B. Fang Z. (2016). Recent developments in film and gas research in modified atmosphere packaging of fresh foods. *Crit. Rev. Food Sci. Nutr.* 56 2174–2182. 10.1080/10408398.2013.819794 25751256

[B54] Zheng X. Huang F. Chen L. Huang L. Cao S. Ma X. (2019). Preparation of transparent film via cellulose regeneration: correlations between ionic liquid and film properties. *Carbohydr. Polym.* 203 214–218. 10.1016/j.carbpol.2018.09.060 30318206

[B55] Zhu S. Wu Y. Chen Q. Yu Z. Wang C. Jin S. (2006). Dissolution of cellulose with ionic liquids and its application: a mini-review. *Green Chem.* 8 325–327. 10.1039/b601395c

[B56] Zong Y. (2007). Research advance of storage technology of mango. *Storage Process* 42 5–8.

[B57] Zorec M. Vodovnik M. Marinšek-Logar R. (2014). Potential of selected rumen bacteria for cellulose and hemicellulose degradation. *Food Technol. Biotechnol.* 52 210–221.

