# Supplementary material for: Biodegradable Cellulose Film Prepared From Banana Pseudo-Stem Using an Ionic Liquid for Mango Preservation
Source: Front Plant Sci. 2021 Feb 19;12:625878. doi: 10.3389/fpls.2021.625878 (PMC7933007; doi:10.3389/fpls.2021.625878)
Supplement: Supplementary file 1 [file Data_Sheet_1.DOCX]

**Biodegradable** **cellulose film prepared from banana pseudo-stem using an ionic liquid for mango preservation**

**Binling Ai^1,2*^, Lili Zheng^1^, Wenqi Li^2^, Xiaoyan Zheng^1^, Yang Yang^1^, Dao Xiao^1^, Jian Shi^2^, Zhanwu Sheng^1*^**

^1^Haikou Experimental Station, Chinese Academy of Tropical Agricultural Sciences, Haikou, Hainan 571101, China

^2^Biosystems and Agricultural Engineering, University of Kentucky, Lexington, KY 40546, USA

*** Correspondence:**Binling Ai, [aibinling@catas.cn](mailto:aibinling@catas.cn);

Zhanwu Sheng, shengz@catas.cn

Table **S1** Comparison of preservation effects of cellulose film and polyethylene film on mango storage at room temperature and 11 °C

| Preservation temperature | Packaging materials | Marketable fruit rate, % | | Diseased fruit rate, % | | Disease index | | Color index | | Weight loss, % | |
| --- | --- | --- | --- | --- | --- | --- | --- | --- | --- | --- | --- |
|  |  | 7 d | 14 d | 7 d | 14 d | 7 d | 14 d | 7 d | 14 d | 7 d | 14 d |
| Room temperature | No packaging | 10 | 0 | 90 | 100 | 5.25 | 10.00 | 7.00 | 10.0 | 11.2±1.0Aa^#^ | 18.9±1.5Ba |
|  | PE film | 0 | 0 | 100 | 100 | 8.50 | 10.00 | 8.25 | 10.0 | 2.0±0.1Ae | 3.5±0.5Bd |
|  | Cellulose film | 80 | 10 | 50 | 90 | 0.50 | 8.25 | 2.00 | 4.5 | 4.1±0.3Ac | 7.6±0.6Bc |
| 11℃ | No packaging | 100 | 70 | 60 | 80 | 1.0 | 2.75 | 1.25 | 1.75 | 7.1±0.5Ab | 13.2±1.5Bb |
|  | PE film | 100 | 50 | 40 | 90 | 1.0 | 3.50 | 1.25 | 3.00 | 1.5±0.2Af | 2.2±0.2Be |
|  | Cellulose film | 100 | 80 | 30 | 50 | 0.5 | 2.50 | 1.50 | 1.75 | 3.0±0.3Ad | 6.3±0.7Bc |

^#^Values are expressed as mean±SEM (n=10). Values in the same row followed by the same uppercase letter are not significantly different at P = 0.05, according to Duncan’s multiple range test. Values in the same column followed by the same lowercase letter are not significantly different at P = 0.05, according to Duncan’s multiple range test.

Table **S2** Weight changes during 4-week periods in soil-buried cellulose film, polyethylene film, and filter paper

|  | PE | Filter paper | Cellulose flim |
| --- | --- | --- | --- |
| Week 1 | 100.1±1.1 Aa^#^ | 95.8±1.3 Ba | 93.9±1.2 Ba |
| Week 2 | 99.1±1.5 Aa | 77.3±5.1 Bb | 70.9±3.2 Bb |
| Week 3 | 99.4±1.7 Aa | 44.2±6.0 Bc | 27.5±6.7 Cc |
| Week 4 | 100.3±1.2 Aa | 15.2±2.1 Bd | 5.7±1.4 Cd |

^#^Values are expressed as mean±SEM (n=3). Values in the same row followed by the same uppercase letter are not significantly different at P = 0.05, according to Duncan’s multiple range test. Values in the same column followed by the same lowercase letter are not significantly different at P = 0.05, according to Duncan’s multiple range test.
